# Facility-Associated Release of Polioviruses into Communities—Risks for the Posteradication Era

**DOI:** 10.3201/eid2507.181703

**Published:** 2019-07

**Authors:** Ananda S. Bandyopadhyay, Harpal Singh, Jacqueline Fournier-Caruana, John F. Modlin, Jay Wenger, Jeffrey Partridge, Roland W. Sutter, Michel J. Zaffran

**Affiliations:** Bill & Melinda Gates Foundation, Seattle, Washington, USA (A.S. Bandyopadhyay, J.F. Modlin, J. Wenger, J. Partridge);; World Health Organization, Geneva, Switzerland (H. Singh, J. Fournier-Caruana, R.W. Sutter, M.J. Zaffran);; Centers for Disease Control and Prevention, Atlanta, Georgia, USA (R.W. Sutter)

**Keywords:** reemerging infectious disease, accidental release, biological, laboratory infections, containment, poliovirus, viruses, vaccine-preventable diseases, polio, poliomyelitis

## Abstract

The Global Polio Eradication Initiative continues to make progress toward the eradication target. Indigenous wild poliovirus (WPV) type 2 was last detected in 1999, WPV type 3 was last detected in 2012, and over the past 2 years WPV type 1 has been detected only in parts of 2 countries (Afghanistan and Pakistan). Once the eradication of poliomyelitis is achieved, infectious and potentially infectious poliovirus materials retained in laboratories, vaccine production sites, and other storage facilities will continue to pose a risk for poliovirus reintroduction into communities. The recent breach in containment of WPV type 2 in an inactivated poliovirus vaccine manufacturing site in the Netherlands prompted this review, which summarizes information on facility-associated release of polioviruses into communities reported over >8 decades. Successful polio eradication requires the management of poliovirus containment posteradication to prevent the consequences of the reestablishment of poliovirus transmission.

In 1988, the World Health Assembly resolved to eradicate poliomyelitis by the year 2000 (*1*). Much progress has been made toward this goal. Two of the 3 wild poliovirus (WPV) serotypes are either certified as eradicated (WPV type 2 [WPV2]) or have not been detected globally since 2012 (WPV type 3 [WPV3]). WPV type 1 (WPV1) continues to circulate only in parts of Afghanistan and Pakistan (*2*). The eradication goal appears within reach.

Once the eradication of poliomyelitis is achieved, apart from rare cases of poliovirus excretors among immunodeficient persons (*3*,*4*), polioviruses will only exist as virus stocks, cultures, and reagents in laboratories, vaccine production sites, and other facilities where live poliovirus stocks are maintained. An essential consideration for the certification of eradication of poliomyelitis as described in the Polio Eradication and Endgame Strategic Plan 2013–2018 (*5*) and the Global Action Plan for Poliovirus Containment (GAPIII) (*6*) is the safe and secure containment of poliovirus within facilities designated by their governments for the posteradication retention of poliovirus materials. The declaration of the certification of WPV2 eradication in September 2015 (*7*) accelerated the implementation of containment work as described in GAPIII. The withdrawal of Sabin poliovirus type 2 from the oral poliovirus vaccine (OPV) in April 2016 highlighted the importance of containment, and annual meetings of the Global Certification Commission (GCC) for the Eradication of Poliomyelitis beginning in September 2015, and especially in October 2017, clarified the GCC oversight responsibilities for containment (*8*,*9*).

A laboratory accident involving the release of WPV2 from an inactivated poliovirus vaccine (IPV) manufacturing site in the Netherlands in April 2017 (*10*–*12*) motivated this historical review to describe the frequency and consequences of similar breaches. Our objective is to remind management and workers at all laboratory and manufacturing facilities of their responsibility to assess the risks of stored materials (*13*).

## Methods

We performed a literature search by using PubMed with no date restrictions, applying the following search terms in various combinations to identify episodes of facility-associated strains infecting humans or being isolated from environmental samples in nonendemic and nonoutbreak reporting areas to complement the information known to us: poliovirus or polio, contamination, accidental environmental contamination, accidental release, and laboratory accidents. This search found a total of 29 references, all of which are described in this article.

## Results

### Major Documented Events

Facility-associated release of polioviruses resulting from either laboratory or vaccine production sources was not uncommon in the period before the development and widespread use of poliovirus vaccines (*14*–*20*) (Tables 1, 2; Figure). In 1933, a 29-year-old physician conducting experimental work on poliomyelitis was bitten by a macaque monkey. Although the exposure to poliovirus could not be confirmed, the physician later experienced paralysis and died (*20*,*21*). The first case of known exposure to poliovirus in a laboratory setting was reported in 1941 in a technician handling infected tissues in preparation for inoculation into monkeys (*19*,*20*,*23*). Six additional laboratory-associated releases of poliovirus through an infected worker occurred during the same decade: 3 in the United States, of which 2 involved a worker infected with Lansing (Armstrong) strain virus (*18*–*20*,*24*,*36*); and 1 each in Zimbabwe (formerly Rhodesia) (*20*,*37*), Canada (*20*,*25*), and the United Kingdom (*20*,*26*). Cases of poliomyelitis attributable to clinical trial use of vaccines or faulty production have also been reported. In 1935, twelve cases of paralytic poliomyelitis, of which 6 were fatal, were reported among those receiving trial vaccinations against poliomyelitis (*22*). In 1955, distribution of 120,000 doses of IPV that had been inadequately inactivated during the production step resulted in the paralysis of 51 children, 5 of whom died; secondary transmission was reported among 113 contacts who experienced paralysis, 5 of whom died (*27*,*28*). Although the 1955 incident was inherently distinct from all other examples discussed in this review (with the root cause being faulty production procedure instead of accidental release or exposure), we include it in this report for completeness. The number of subclinical infections with poliovirus during this period is unknown, so the total number of persons affected might have been many times higher (*29*).

**Table 1:** Reported incidents of poliovirus release from laboratories and vaccine production facilities in the pre–polio vaccine era*

Year	Location (reference)	Source	Poliovirus type	No. cases*	Exposure			Description
					Primary	Secondary	Tertiary	
1933	United States (*20*,*21*)	Lab	Not indicated	1	Physician	NA	NA	Bitten (cutaneous disruption) by a normal macaque while doing work on poliomyelitis (paralysis); filterable virus capable of reproducing the disease in rabbits was isolated from the case; case was fatal
1935	United States (*22*)	Vaccine production facility	Not indicated	12	Vaccine trial patients, age 5 mo to 20 y	NA	NA	12 cases of paralytic poliomyelitis in patients receiving trial vaccination against poliomyelitis; natural infections ruled out as cause; 6 deaths
1941	United States (*19*,*20*,*23*)	Lab	Not indicated	1	Lab staff	NA	NA	Lab staff member experienced paralysis after preparation of infected tissues for inoculation into monkeys; cutaneous inoculation; no polio outbreaks reported in place of residence or areas of travel
1945	United States (*20**,**23*)	Lab	Not indicated	1	Lab staff	NA	NA	Lab staff member scratched on hand by noninoculated monkey during transport; subsequent virus contamination of hands might have occurred while feeding or inoculating monkeys; patient experienced paralysis and later died
1946	Zimbabwe (formerly Rhodesia) (*20**,**25*)	Lab	Not indicated	1	Lab staff	NA	NA	Infection acquired during inoculation of monkeys with polio virus; paralysis occurred, case was fatal
1949	United States (*20*,*24*)	Lab	WPV2 (mouse- adapted Lansing strain)	2	Lab staff	NA	NA	Two lab technicians were infected in the eyes and nose with Lansing (Armstrong) strain while inoculating mice during polio experiments; both experienced paralysis
1950	Canada (*20*,*25*)	Lab	Not indicated	1	Physician	Na	NA	Doctor acquired poliomyelitis while performing autopsy on poliomyelitis patient; intracutaneous inoculation; residual weakness; case was not fatal
1954	United Kingdom (*20*,*26*)	Lab	WPV2 (MEF-1) strain	1	Lab staff	NA	NA	Lab technicians infected by cutaneous inoculation while performing necropsy on animals infected with wild type-2 (MEF-1) strain; subsequent paralysis; cases were not fatal

*Cases defined as laboratory positive (with or without paralysis) for poliovirus by standard methods of virus isolation or known exposure to poliovirus. Lab, laboratory; MEF-1, wild poliovirus type 2 laboratory reference strain; NA, not applicable; WPV2, wild poliovirus type 2.

**Table 2:** Reported incidents of poliovirus release from laboratories and vaccine production facilities in the post–polio vaccine era*

Year	Location (reference)	Source	Poliovirus type	No. cases	Exposure			Description
					Primary	Secondary	Tertiary	
1955	United States (*27*,*28*)	Vaccine production facility	Not indicated	164	Vaccine recipients(≈40,000 children)	113 contacts of the children	NA	“Cutter incident”; inadequate formaldehyde virus inactivation during poliovirus vaccine production (≈120,000 doses); ≈40,000 children experienced muscle weakness, of whom 51 experienced paralysis; 5 deaths; 113 contacts of the children were also paralyzed, of whom 5 died
1991	France (*29*)	Lab and vaccine production facility	WPV3 (Saukett) strain	1	No definitive information on exposure of case			Saukett strain isolated in France from a woman from Algeria; source of this lab strain could not be confirmed
1992	Netherlands (*30*)	Vaccine production facility	WPV1 (Mahoney) strain	1	Father (worker at facility)	Son	NA	Boy (age 19 mo) with respiratory symptoms (no paralysis); father with history of accidental exposure to Mahoney strains while working in a poliovirus vaccine production facility
1993	Netherlands (*30*)	Vaccine production facility	WPV3 (Saukett) strain	1	No definitive information on exposure of case			Child with gastroenteritis (no paralysis); had travel history to France; no epidemiology established to trace lab exposure; Saukett strains typical for IPV production in France isolated from the stool samples
2000	India (*31*–*33*)	Lab and vaccine production facility	WPV2 (MEF-1) strain	3	No definitive information on exposure of case			WPV2 isolates found in Sep 2000 and Nov 2002–Feb 2003 from 10 children with AFP, 1 healthy contact, and 1 environmental sample; isolates unrelated to all previous WPV2 strains found in India; because this was a lab reference strain and not a community-derived wild strain, lab source was suspected
2002–2003	India (*31*–*33*)	Lab and vaccine production facility	WPV2 (MEF-1) strain	8	No definitive information on exposure of case			
2014	Belgium (*34*,*35*)	Vaccine production facility	WPV3 (Saukett) strain	0	NA	NA	NA	≈10^13^ infectious WPV3 particles accidentally released into sewage system from production plant in Belgium; no poliovirus detected in environmental or human samples
2017	Netherlands (*12*)	Vaccine production facility	WPV2 (MEF-1) strain	1	Worker	None	None	Accidental leakage in vaccine production room; 1 of 2 exposed staff members tested positive by RT-PCR

*Cases are defined as laboratory positive (with or without paralysis) for poliovirus by standard methods of virus isolation or known exposure to poliovirus. AFP, acute flaccid paralysis; IPV, inactivated poliovirus vaccine; lab, laboratory; MEF-1, wild poliovirus type 2 laboratory reference strain; NA, not applicable; RT-PCR, reverse transcription PCR; WPV, wild poliovirus; WPV1, wild poliovirus type 1; WPV2, wild poliovirus type 2; WPV3, wild poliovirus type 3.

**Figure:**
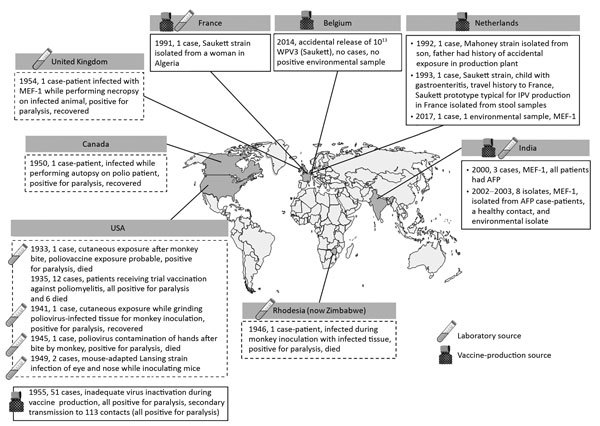
Reported incidents of facility-associated poliovirus release from laboratories and manufacturing sites in the pre–polio vaccine era (shown inside dashed-line frames) and the time of poliovirus vaccine introduction to the present (shown inside solid-line frames). AFP, acute flaccid paralysis; IPV, inactivated poliovirus vaccine; MEF-1, wild poliovirus type 2 laboratory reference strain; WPV, wild poliovirus; WPV3, wild poliovirus type 3.

In the 3 decades since the WHA resolution to eradicate poliomyelitis and the formation of the Global Polio Eradication Initiative (GPEI) in 1988, seven documented incidents underscore the potential for facility-associated release of polioviruses into the community in the modern era. In 1991, a WPV3 (Saukett strain), probably from a laboratory source, was isolated in France from a woman from Algeria. A year later, a worker in a vaccine manufacturing facility in the Netherlands transmitted a WPV1 (Mahoney strain) used for IPV production to his son (*30*). In another incident in the Netherlands in 1993, a child with a travel history to France was reported to have been infected with a strain of WPV3 (Saukett strain) almost identical to that used for IPV production in France. The possibility of laboratory contamination was ruled out, and environmental samples collected from around the child’s home and among his family contacts were negative for poliovirus in cell culture. The source of this infection was not determined (*30*). In India, 2 incidents were reported during 2000–2003 after the interruption of WPV2 transmission in 1999. A WPV2 laboratory reference strain (MEF-1) was recovered from 3 poliomyelitis patients in September 2000 and 7 patients during November 2002–February 2003. The sources of these infections were not identified (*31*–*33*).

In September 2014 in Belgium, ≈10^13^ infectious WPV3 particles were released into the sewage system from a vaccine production plant (*34*). Subsequent investigations revealed no evidence of WPV3 in samples from a range of environmental samples (*35*). More recently, in the Netherlands, WPV2 (MEF-1 strain) was accidentally released as an aerosolized high-titer spill when tubing became disconnected in a vaccine production room. One exposed staff member became infected and shed the wild virus strain for ≈4 weeks before testing negative by fecal culture, whereas a second staff member who was also present at the time of the spill did not test positive for poliovirus in throat swabs or stool samples (*12*). No acute flaccid paralysis (AFP) cases or secondary spread to household contacts was detected.

## Discussion

The laboratory accident in April 2017 involving the exposure of a worker who subsequently excreted WPV2 into the sewage system (*12*) serves as a stark reminder that the achievements of the GPEI are fragile and can be reversed if remaining sources of polioviruses are not contained to reduce the likelihood and consequences of virus reintroduction after eradication. Even more recently, a suspected contamination of ≈150,000 bivalent OPV vials with type 2 Sabin virus strains has been reported in India (*38*), highlighting the importance of completeness of containment-related activities for type 2 OPV at all stages of vaccine manufacturing and release. Our review demonstrates that the known risk for poliovirus release from a facility into the community appears to be small, based on only a handful of reported incidents, primarily from vaccine manufacturing sites. However, these reported incidents might represent the proverbial tip of the iceberg, given that the reporting requirements in the past were not very stringent.

The release of polioviruses into the community in the posteradication era is a major public health concern for GPEI as it implements the planned, sequential cessation of OPV use during the polio endgame. With the global discontinuation of type 2 OPV for routine and supplemental immunization in April 2016 and the planned cessation of all OPV use in the next 4–5 years, such release of polioviruses from facilities into the community will become a greater public health concern because population immunity wanes in settings of high population density, poor hygiene, and suboptimal immunity (e.g., tropical developing countries) after OPV withdrawal, increasing the potential for transmission (*39*).

We should note that in the prevaccine era most of the reported incidents occurred in research settings and the exposed persons did not have vaccine-induced immunity for protection against paralysis or virus transmission. In sharp contrast, the incidents reported in the past 3 decades were related to containment failures at vaccine production plants where the staff are expected to be fully vaccinated. Incidents in the more recent period are likely to be more representative of the public health impact of such containment failures for the current phase of the polio endgame and the near future. 

The paucity of reports of laboratory-associated poliomyelitis during the past 3 decades testifies to the effectiveness of vaccines and to improved laboratory facilities and biorisk management. However, laboratory breaches in the past might not have been recognized in the absence of clinical cases, and environmental surveillance was less extensive than it is today. Environmental surveillance for polio, for example, appears to have played a major role in such incidents primarily in the past 2 decades, and more so in recent times, with the expansion and enhancement of scope and methodologies that were introduced as a component of the polio endgame. Furthermore, reporting requirements were less stringent in the past, and we assume that not all facility incidents were recorded. The lack of documented incidents of laboratory accidents during 1955–1991 is difficult to explain but might also have been affected by these factors, in addition to the fact that the formation of GPEI in 1988 led to a more concerted, globally synchronized effort to track and report polio cases and outbreaks. 

As seen in the Netherlands incident in 2017, previously vaccinated persons, although probably protected from paralysis, can excrete poliovirus after accidental exposure from containment failures and put the community at risk for virus transmission. This risk underscores the importance of stringent containment measures at the vaccine production sites and preparedness for deployment or enhancement of surveillance activities, such as environmental monitoring, as a public health response strategy.

The smallpox experience illustrates the importance of containment for an eradicated pathogen. Within 1 year of detection of the last known natural case of smallpox in 1977, a case linked to laboratory transmission was reported (*40*). We have a historic opportunity to benefit from an additional 40 years of experience in risk management and disease-control measures to ensure containment measures are designed, implemented, and maintained to provide a world free from all risks for polio-related paralysis. The first step is a uniform, global awareness of the importance of implementing the GAPIII guidelines to minimize facility-associated risks for poliovirus reintroduction. The risk for accidental release can be minimized by retaining poliovirus in a limited number of poliovirus-essential facilities. GAPIII also proposes further risk reduction by establishing international standards of biorisk management for facility containment (i.e., primary safeguards), population immunity (i.e., secondary safeguards), and facility location (i.e., tertiary safeguards) with assurance by national (National Authority for Containment) and international (GCC) oversight that such standards are met. Timeliness and completeness in implementing these measures through well-defined risk management systems and an effective National Authority for Containment are key for success in sustaining a polio-free world.

Equally important is the identification of materials that are potentially infectious for polioviruses in all facilities that store human stool specimens, respiratory samples, or environmental sewage for any purpose. Depending on the place and time of collection, such materials might harbor infectious polioviruses that have been eradicated (WPV2) or are nearly eradicated (WPV1 and WPV3) in the wild. Identifying the risk, eliminating the risk through destruction, or mitigating the risk of handling such materials is essential (*13*).

Finally, the risk management approach to containment might have to be optimized and balanced to enable other risk mitigation efforts for the endgame and beyond, such as novel vaccine and drug development to further reduce any risk for vaccine-derived circulation. Timely availability of antiviral drugs and effective novel OPVs that have less risk for reversion to neurovirulence compared with current Sabin vaccines might strengthen outbreak response strategies and mitigate medical risks for inadvertent poliovirus exposure and improve reporting of accidental safety breaches by employees (*41**–**43*). The World Health Assembly resolution on poliovirus containment (WHA71.16) urges intense efforts to accelerate progress toward poliovirus containment globally (*44*).
